# Case of Twins

**Published:** 1845-06

**Authors:** 


*Case of Twins; there being an interval of thirty-two days between their Birth.*—John Irvine, a surgeon in the British navy, records, in the *Medical Times*, (28 Dec., 1844,) the following remarkable case. Mary F., aged 35 years, was seized with labour pains, on the 1st of October, at the full period of utero-gestation; and was delivered by Dr. Burleigh, of a healthy, but rather small-sized female infant. Three hours afterwards the placenta was cast off by the natural contraction of the uterus. Dr. Burleigh, on examining the patient at this stage, clearly ascertained that the uterus was still in a gravid state. After waiting some time for the renewal of parturient efforts, he left, with instructions to be sent for when labour commenced. On visiting his patient three days afterwards, he found her out of bed following her usual domestic affairs, and in good health. On the 2d of the following November labour pains came on, and just as Dr. Burleigh arrived, a large healthy male infant was born, and two hours afterwards Dr. B. extracted the placenta. The patient's recovery was as rapid as previously, for on the third day she was attending to her family concerns.—*Am. Jour. Med.*

